# Machine Learning Models Decoding the Association Between Urinary Stone Diseases and Metabolic Urinary Profiles

**DOI:** 10.3390/metabo14120674

**Published:** 2024-12-03

**Authors:** Lin Ma, Yi Qiao, Runqiu Wang, Hualin Chen, Guanghua Liu, He Xiao, Ran Dai

**Affiliations:** 1 Department of Urology, Peking Union Medical College Hospital, Chinese Academy of Medical Sciences and Peking Union Medical College, Beijing 100730, China; malin55@pumch.cn (L.M.); qiaoyi@pumch.cn (Y.Q.); chenhualin511@gmail.com (H.C.); liugh@pumch.cn (G.L.); 2 Department of Biostatistics, College of Public Health, University of Nebraska Medical Center, Omaha, NE 68198, USA; ruwang@unmc.edu

**Keywords:** machine learning, urinary stone diseases, metabolites, biomarkers, random forest

## Abstract

**Background:** Employing advanced machine learning models, we aim to identify biomarkers for urolithiasis from 24-h metabolic urinary abnormalities and study their associations with urinary stone diseases. **Methods:** We retrospectively recruited 468 patients at Peking Union Medical College Hospital who were diagnosed with urinary stone disease, including renal, ureteral, and multiple location stones, and had undergone a 24-h urine metabolic evaluation. We applied machine learning methods to identify biomarkers of urolithiasis from the urinary metabolite profiles. In total, 148 (34.02%) patients were with kidney stones, 34 (7.82%) with ureter stones, and 163 (34.83%) with multiple location stones, all of whom had detailed urinary metabolite data. Our analyses revealed that the Random Forest algorithm exhibited the highest predictive accuracy, with AUC values of 0.809 for kidney stones, 0.99 for ureter stones, and 0.775 for multiple location stones. The Super Learner Ensemble Method also demonstrated high predictive performance with slightly lower AUC values compared to Random Forest. Further analysis using multivariate logistic regression identified significant features for each stone type based on the Random Forest method. **Results:** We found that 24-h urinary magnesium was positively associated with both kidney stones and multiple location stones (OR = 1.195 [1.06–1.3525] and 1.3258 [1.1814–1.4949]) due to its high correlation with urinary phosphorus, while 24-h urinary creatinine was a protective factor for kidney stones and ureter stones, with ORs of 0.9533 [0.9117–0.996] and 0.8572 [0.8182–0.8959]. eGFR was a risk factor for ureter stones and multiple location stones, with ORs of 1.0145 [1.0084–1.0209] and 1.0148 [1.0077–1.0223]. **Conclusion:** Machine learning techniques show promise in revealing the links between urological stone disease and 24-h urinary metabolic data. Enhancing the prediction accuracy of these models leads to improved dietary or pharmacological prevention strategies.

## 1. Introduction

Urolithiasis, characterized by the formation of stones in the urinary tract, is a prevalent condition that poses significant clinical challenges [1]. The treatment approach for urolithiasis is highly dependent on the type of stone involved—whether calcium oxalate, uric acid, or struvite stones—each necessitating distinct therapeutic strategies [2]. Despite advances in treatment, accurate prediction and early diagnosis remain critical for the effective management and prevention of recurrence [3]. Current clinical challenges in diagnosing and managing urinary stones include limitations in early detection, frequent recurrence, and variability in treatment outcomes. These issues are compounded by the need for the accurate identification of underlying metabolic disorders contributing to stone formation. Dietary intake significantly impacts urinary metabolite levels, as the kidneys filter and excrete substances derived from food and beverages. For instance, dietary sodium, calcium, and magnesium intake are closely associated with urinary excretion of these ions. This relationship underscores the importance of evaluating dietary habits when interpreting urinary metabolic profiles in urolithiasis patients. Therefore, our study aims to leverage 24-h urinary metabolite data not only to identify biomarkers but also to infer potential dietary contributions to stone formation, which may inform personalized dietary interventions.

Recent advances in biomedical research have increasingly leveraged machine learning (ML) to enhance diagnostic accuracy and treatment planning across various medical conditions [4,5,6]. Although imaging-based results from ultrasound are the gold standard for the diagnosis of urinary stones, metabolite-based biomarker development offers great advantages in allowing for early detection and intervention, cost-effective monitoring, and shorter turnaround time. Our research aims to harness these advantages by employing advanced ML models to identify potential biomarkers from 24-h metabolic urinary profiles. The diagnosis and localization of urinary stones in patients mainly rely on the results of the most current imaging examinations, including CT, ultrasound, or X-ray. All imaging findings were diagnosed by specialists of radiology. While individual biomarkers may offer limited predictive power, their collective analysis through ML algorithms can significantly enhance the accuracy of diagnosing, predicting, and monitoring urolithiasis [7]. Machine learning offers unique value in this context by revealing intricate patterns within metabolite profiles that may not be apparent through traditional analysis, thereby enhancing predictive accuracy and enabling more personalized patient management. We hypothesize that urinary metabolites, when analyzed through machine learning models, can serve as reliable predictors for different stone types, thereby offering a potential foundation for individualized treatment strategies.

By systematically analyzing complex data from 24-h urine collections, which encompass a wide range of metabolites, our ML models aim to refine the prediction of different types of urinary stones [8]. This approach not only improves early diagnostic precision but also uncovers intricate patterns and associations between metabolic biomarkers and urolithiasis that traditional analytical methods may overlook [9].

The objective of this research is two-fold: first, to develop robust predictive models based on multivariate metabolic abnormalities in urine specific to various stone types, and second, to elucidate the underlying associations between these metabolic profiles and urolithiasis [3]. Through this integrative approach, we seek to create more precise early diagnostic tools that can inform personalized treatment plans, ultimately enhancing patient outcomes in the management of urolithiasis. This study advances the field by merging cutting-edge machine learning techniques with clinical practice to address a common yet complex medical condition [10].

## 2. Methods

### 2.1. Data Description and Preparation

From January 2013 to April 2019, we retrospectively recruited 468 patients at our institution who were diagnosed with urinary stone disease, including renal, ureteral, and bladder stones and had undergone a 24-h urine metabolic evaluation. The dataset comprises baseline demographic, comorbidity, and metabolite information for 468 patients. Baseline demographic data encompass gender, age, and body mass index (BMI). Comorbidity profiles include high blood pressure (HBP), high cholesterol, diabetes, hyperuricemia, hyperparathyroidism, and uric acid levels. Information on metabolites consists of estimated glomerular filtration rate (eGFR); urinary excretion rates per 24-h for oxalate, citrate, calcium, uric acid, creatinine, phosphorus, and magnesium; daily sodium intake based on 24-h urinary sodium excretion; urine pH; and total urine volume over 24-h. The outcome variables are focused on stones; we are interested in kidney stones, ureter stones, and multiple location stones. Our goal is to identify risk predictors from the list of candidate variables, including baseline demographic, comorbidity, and metabolite information, to predict kidney stones, ureter stones, and multiple location stones (i.e., the co-existence of kidney stones and ureter stones).

### 2.2. Machine Learning Models

In order to achieve both goals for prediction and risk predictor identification from the metabolic data, a collection of supervised models are used to build the predictive models in this paper, including stepwise logistic regression, logistic regression with least Absolute Shrinkage and Selection Operator (Lasso), Random Forest, XGBoost, and the Super Learner Ensemble Method.

#### 2.2.1. Stepwise Based on Logistic Regression

Stepwise regression [11] involves iteratively adding or removing predictors based on specific criteria such as statistical significance (we use *p* ≤ 0.05 in this paper), AIC (Akaike Information Criterion) [12], or BIC (Bayesian Information Criterion) [13]. By excluding irrelevant predictors, the model can potentially perform better on new, unseen data. In this paper, we apply the bidirectional stepwise regression with the AIC criterion:

$$AIC=2k-2\ln\left(\hat{L}\right),$$

where *k* is the number of estimated parameters in the model, and $\hat{L}$ is the maximized value of the likelihood function for the model.

#### 2.2.2. Lasso Based on Logistic Regression

The least Absolute Shrinkage and Selection Operator (Lasso) is a parametric method of feature selection used to delete redundant features. The main idea of Lasso is to shrink some coefficients to zero by adding a penalty [14]. As a result, Lasso can use those important features to build a regression model. Because of the binary outcomes of this paper, a logistic regression with important features selected by Lasso was conducted.

$${\hat{\beta }}_{Lasso}={\arg min}_{\beta }\sum_{i=1}^{n}\left[ln(1+exp(x_{i}\beta \right))-y_{i}x_{i}\beta ]+\lambda |\beta |$$

where the first term is the negative log likelihood for the logistic regression and the second term is the penalized term. The tuning parameter λ is chosen using 10-fold cross-validation in this paper.

#### 2.2.3. Random Forest

Random Forest is a supervised method that applies a bootstrap algorithm to randomly select subset samples with replacement and use them to build multiple decision trees [15]. Random Forest combines the classification of each decision tree, and the final classification result depends on the vote of each decision tree.

In addition, Random Forest can quantify the importance of each feature based on the Gini Index [16]. Gini impurity is calculated to decide which variable to split at each node. For each variable, the sum of the Gini decreases across every tree of the forest and is accumulated every time that variable is chosen to split a node. The average of Gini is obtained by the sum dividing the number of trees. In this paper, the number of trees is set to 500.

#### 2.2.4. XGBoost

XGBoost, short for eXtreme Gradient Boosting, is also a tree-based machine learning algorithm [17]. It is an innovative sparsity-aware algorithm tailored for handling sparse data and a weighted quantile sketch to facilitate approximate tree learning. XGBoost effectively manages datasets with billions of examples, utilizing significantly fewer resources compared to existing systems.

#### 2.2.5. Super Learner Ensemble Method

The Super Learner ensemble is a sophisticated machine learning method designed to enhance prediction accuracy by combining multiple algorithms into a single predictive model [18]. This approach integrates the strengths of both parametric models, such as linear regression and non-parametric models, such as Random Forest and neural networks. By aggregating the predictions of diverse algorithms, Super Learner can capture more complex data patterns and improve overall performance.

### 2.3. Evaluation Methods

A confusion matrix was used in this study to calculate the accuracy, precision, recall, F1-score, and area under the curve (AUC) (Table 1). Accuracy measures the overall correctness of the model’s predictions, while precision quantifies the proportion of true positive predictions among all positive predictions made by the model. Recall evaluates the model’s ability to correctly identify all true positives, and the F1-score provides a balanced measure that considers both precision and recall. AUC measures the area under the ROC (received operating curve) curve. ROC is a visualization tool that accesses the diagnostic ability of binary outcomes with varying discrimination thresholds [19,20]. Each of these metrics range from 0 to 1, with values closer to 1 indicating better model performance and fit.

$$Accuracy=\frac{TP+TN}{TP+FP+TN+FN}$$

$$Precision=\frac{TP}{TP+FP}$$

$$Recall=\frac{TP}{TP+FN}$$

$$F_{1}=2\times \frac{Precision*Recall}{Precision+Recall}$$

### 2.4. Data Modeling

In order to achieve this goal, data pre-processing was first conducted. We check the missingness and remove predictors with more than 30% of missing values. For predictors with less than 30% of missing values, multiple imputations were performed. We also checked the co-linearity between continuous predictors and removed the independent variable (we deleted Serum Creatinine) with the co-linear issue (Figure S1 in Supplementary Materials). Second, univariate analysis was performed to explore important predictors that show significant differences between stone patients and healthy patients (Table 2, Tables S1 and S2). Third, stepwise logistic regression and supervised machine learning models, such as Lasso, Random Forest, and XGBoost, along with the Super Learner ensemble, which aggregates these methods, are employed to predict the probability of different types of urinary stones. Fourth, to minimize the risk of overfitting, we partitioned the data, using 75% for training and 25% for testing. We compare the performances for the testing date among the above models and qualify the importance of variables based on the model with the highest performances. Fifth, training and testing datasets were imputed separately by using multiple imputations, and predictive mean matching (PMM) was performed with M = 5 imputations. Sixth, to address the issue of outcome imbalance, the Synthetic Minority Oversampling Technique (SMOTE) [21] was applied to train data to handle the class imbalance. Finally, to explore the interaction term, we used Lasso to select the interaction term first and then applied the Random Forest, XGboost, and the Super Learner ensemble method to evaluate the prediction.

Different R packages are used for each method: ‘glmnet’ for Lasso, ‘randomForest’ for the Random Forest, ‘xgboost’ for XGBoost, ‘SuperLearner’ for the combined model, and ‘ROCR’ for ROCs. Tuning parameters include Regularization Strength (lambda) for Lasso; number of trees (ntree) and number of variables randomly selected at each split for Random Forest; number of boosting iterations (nrounds), learning rate (eta), maximum depth of each tree (max_depth), minimum loss reduction to make a split (gamma), fraction of samples used for each tree (subsample), fraction of features to consider for each tree (colsample_bytree), and L1 and L2 regularization terms (alpha, lambda) for XGBoost. Ten-fold cross-validation was used to select the tuning parameters. All analysis was conducted by RStudio (version 4.3.1; 2023-0616 RStudio, Inc. Boston, MA, USA).

## 3. Results

### 3.1. Patients’ Characteristics

Table 2 shows the descriptive statistics for candidate risk factors. There are 468 patients collected in total, with 148 kidney stone patients, 34 ureter stone patients, and 163 multiple location stone patients. Thirty-three patients did not answer the questions about kidney stones and ureter stones; we ignored those patients in both studies. The univariate analysis shows that urinary citrate (24H), daily sodium intake, urinary creatinine (24H), and urinary magnesium (24H) have a significant association with kidney stones (*p* < 0.05) (Table 2); diabetes is the only significant factor for ureter stones (Table S1 in Supplementary Materials). eGFR and urinary magnesium (24H) have a significant association with multiple location stones (Table S2 in Supplementary Materials).

### 3.2. Important Features Identified by Machine Learning Techniques

**Figure 1:**
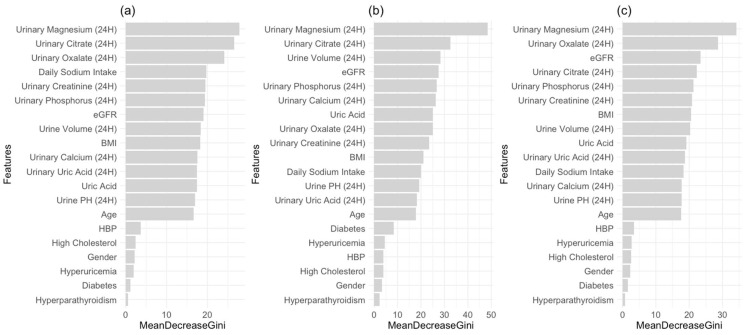
Importance plots from Random Forest of (**a**) kidney stones, (**b**) ureter stones, and (**c**) multiple location stones: the importance of each variable is measured by the mean decrease from one of the five imputed datasets during the multiple imputation.

We apply stepwise regression, Lasso, XGBoost, and Random Forest to show the importance of features. For the outcome kidney stone, the stepwise method selects “HBP”, “Hyperparathyroidism”, “Uric Acid”, “eGFR”, “Urinary.Citrate (24H)”, “Urinary.Calcium (24H)”, “Daily Sodium Intake (24H)”, “Urinary Creatinine (24H)”, and “Urinary Magnesium (24H)”. The Lasso model selects “HBP”, “Hyperparathyroidism”, “eGFR”, “Urinary.Citrate (24H)”, “Daily Sodium Intake (24H)”, “Urinary Creatinine (24H)”, and “Urinary Magnesium (24H)”. As we mentioned before, Random Forest and XGBoost can quantify the importance of each feature. The important plots identified by Random Forest are shown in Figure 1, and the important plots identified by XGBoost are shown in the Supplementary Materials Figure S2.

### 3.3. Sampling Technique Experiment

**Figure 2:**
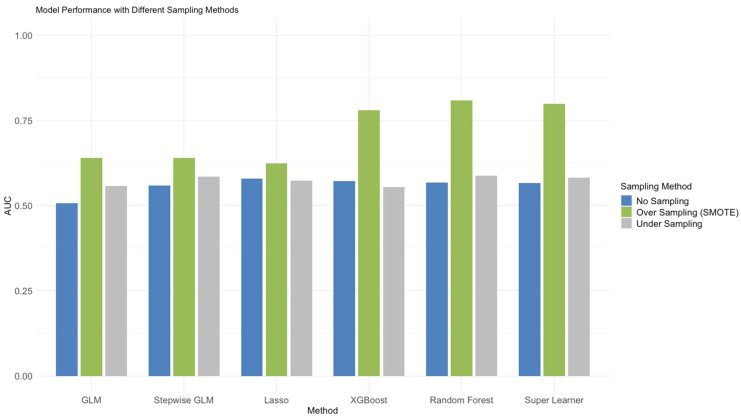
The AUC comparison of sampling techniques in five different models.

Due to the imbalanced data from the urinary stone diseases compared to other groups, the evaluation parameter may not reflect the learning results. We use R package “smotefamily” and “ROSE” to solve the sample imbalance issue. We applied SMOTE as an oversampling method to oversample the minority classes and enhance the performance of the classification models. In our implementation, SMOTE was applied only for training data using five nearest neighbors (K = 5) and a duplication factor of 1 (each minority class instance was oversampled once) for kidney stones, 10 for ureter stones, and 1 for multiple location stones. We also compared the AUC results of oversampling, under sampling, and no sampling (shown in Figure 2). The oversampling method adjusts the AUC to 0.64–0.99, which is higher than that for under sampling and no sampling. Therefore, to further optimize our models, we conducted SMOTE in six different models.

### 3.4. Model Performance Comparison Among Machine Learning Techniques

**Figure 3:**
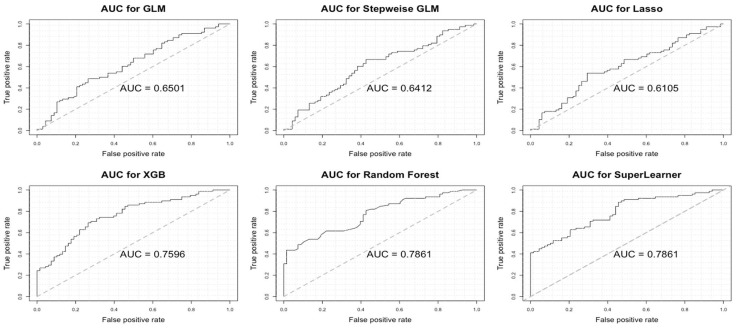
ROC among machine learning techniques (based on one of the five imputed datasets) for the kidney stones.

Table 3 shows the mean of five imputed datasets for model performances among methods including stepwise, Lasso, XGB, Random Forest, and the ensemble algorithm (Super Learner) for kidney stones, ureter stones, and multiple location stones, and Figure 3 shows the ROC for one of the five imputed data. Overall, the Random Forest algorithm has the highest AUCs, followed by XGB. The ensemble algorithm takes advantage of all the algorithms and performs with more stability. Since the combined algorithm will apply the algorithm that has the best performance to show the importance of risk factors, we will use the Random Forest model to select important risk factors. The model performances for the ureter stones and multiple location stones are shown in Supplementary Materials Figures S5 and S6.

### 3.5. Logistic Regression Using the Top-10 Important Risk Factors Identified by Random Forest

Multivariate logistic regression models are conducted by using the top-10 important risk factors identified by Random Forest for three outcomes: kidney stones, ureter stones, and multiple location stones.

The results are presented in Table 4, Table 5 and Table 6. For kidney stones, urinary calcium (24H) shows a significant positive association (OR = 1.1036, 95% CI [1.0373, 1.1757]). Urinary magnesium (24H) also shows a positive association with kidney stones (OR = 1.1950, 95% CI [1.06, 1.3525]). In addition, there is a marginal negative association between daily sodium intake and kidney stones (OR = 0.9051, 95% CI [0.8398, 0.9731]). For ureter stones, urine volume, eGFR, uric acid, urinary oxalate (24H), and BMI exhibit strong associations with ureter stones, while higher urinary creatinine levels are associated with a lower risk of ureter stones (OR = 0.8572, 95% CI [0.8182, 0.8959]). Urinary calcium is also significantly associated with ureter stones (OR = 1.1532, 95% CI [1.0905–1.221]). For multiple location stones, urinary magnesium (OR = 1.3258, 95% CI [1.1814, 1.4949]) and eGFR (OR = 1.0148, 95% CI [1.0077, 1.0223]) show strong positive associations. Urinary citrate (24H) has a significant but weaker association with multiple location stones.

### 3.6. Exploring for the Interaction

To evaluate the effect of interactions, we included 20 variables and all possible two-way interactions in the model and employed Lasso for interaction term selection. Lasso identified only a few interaction terms. Subsequently, we applied XGBoost, Random Forest, and the Super Learner Ensemble Method to the testing data to assess predictive performance. Table 7 shows the results for kidney stones, ureter stones, and multiple location stones, respectively. The results indicate that after including interaction terms, the performance of the models remains comparable to the models without interaction terms (compared with Table 2). Therefore, interaction terms do not appear to play a significant role in improving prediction performance.

### 3.7. Sensitivity Analysis

**Figure 4:**
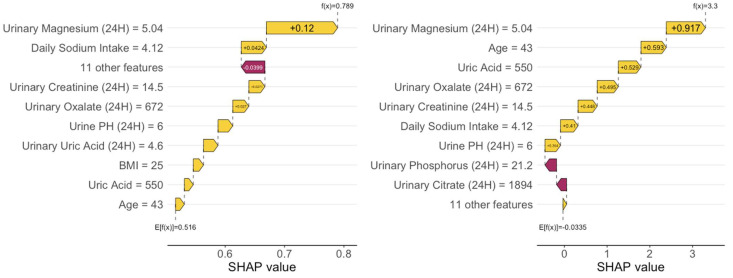
SHAP plots by Random Forest (**left**) and XGBoost (**right**) for predicting kidney stone risk: the SHAP values are calculated from one of the five imputed datasets during the multiple imputation.

As part of the sensitivity analysis for our multivariate logistic regression, we applied SHapley Additive exPlanations (SHAP) [22] to interpret the machine learning models. Figure 4, Figures S3 and S4 show the results for kidney stones, ureter stones, and multiple location stones, respectively. The SHAP analysis confirmed that urinary magnesium (24H) had the largest positive contribution to the prediction of kidney stones, while urinary creatinine (24H) demonstrated a negative contribution to the prediction of ureter stones. Additionally, both urinary magnesium (24H) and eGFR were significant contributors to the prediction of multiple location stones. These findings are consistent with the results obtained from the multivariate logistic regression.

From Figure S1, urinary magnesium (24H) and urinary phosphorus (24H) have a strong positive correlation (Pearson correlation = 0.5). The large correlation could potentially influence the effects of urinary phosphorus in the model. Table 8, Table 9 and Table 10 present the results of the multivariate analysis after excluding urinary magnesium (24H) as a feature (i.e., we removed urinary magnesium (24H) and performed our proposed machine learning algorithms for prediction and used the top-10 variables identified by Random Forest in multivariate logistic regression). The results show that after removing urinary magnesium (24H), urinary phosphorus (24H) become significantly positive associated with kidney stones and multiple location stones. Mechanistic details are discussed in Section 4.

## 4. Discussion

This study uses the 24-h urine metabolic evaluation data to predict urinary stones and further investigates the risk factors associated with different types of kidney stones, specifically kidney stones, ureter stones, and multiple location stones, in a cohort of 468 patients. Through comprehensive statistical analyses, several significant associations have been identified, providing insights into the pathophysiology and potential prevention strategies for these conditions. On the one hand, relevant research results can provide reliable medical evidence for clinical medical and nursing decision-making. On the other hand, they can guide governments and health officials to formulate appropriate health economic policies.

Taking advantage of the machine learning and ensemble learning methods, the prediction accuracy was enhanced, and the importance of the metabolomic predictors was quantified.

Multivariate logistic regression models, using the top-10 important risk factors identified by Random Forest, further refined these associations. For kidney stones, urinary magnesium (24H) remained a significant factor in both univariate and multivariate models, with patients exhibiting higher urinary magnesium levels, showing increased risks. This finding aligns with the existing literature suggesting that magnesium can influence stone formation through its correlations with other urinary constituents. For example, it has been shown in a previous study that the magnesium level in 24-h urinalysis has a direct association with 24-h urine oxalate [23]. In our study, we found that the urinary magnesium level is highly correlated with urinary phosphorus, which is a risk factor of urinary stones (see Section 3.5). When both calcium and phosphate concentrations are high, there is an increased risk of calcium phosphate stone formation. This study also suggests that elevated urinary calcium is a risk factor for kidney stones, which is consistent with previous studies and the consensus that elevated urinary calcium levels contribute to calcium stone formation through supersaturation and crystallization processes [6]. In the case of ureter stones, urinary creatinine and urinary calcium were significant. Higher urinary creatinine levels were associated with a reduced risk, while higher urinary calcium levels increased the risk. This dual effect might be explained by the role of creatinine as a marker of muscle mass and overall health, while elevated urinary calcium is a well-known risk factor for stone formation [24]. In addition, urine volume, eGFR, 24-h urine uric acid, 24-h urine oxalate and BMI were strongly correlated with ureter stones. Previous studies have also suggested that metabolic syndrome, including hyperuricemia and obesity, is highly associated with urinary stones, which can help us understand the relationship between 24-h urinary uric acid and BMI and stones [25,26]. The association between urine volume and ureter stones seems to be contrary to previous studies. In the past, it was generally believed that low urine volume is an important factor in the development of urinary stones [27]. We speculate that the correlation between urine volume and ureter stones found in this study may be related to the greater tendency of kidney stones to move down to the ureter with increased urine volume. However, this is only our conjecture, which needs to be confirmed by further research. For multiple location stones, significant factors included urinary magnesium, eGFR, and urinary calcium. Higher levels of these factors were associated with an increased risk of stone formation, reinforcing the importance of comprehensive metabolic evaluation in patients with recurrent or multiple location stones. Our findings emphasize the critical role of dietary intake in modulating urinary metabolite levels. Elevated urinary calcium and magnesium levels, identified as risk factors for kidney and multiple location stones, may partly reflect high dietary intake of these minerals. Conversely, urinary citrate, a protective factor against stone formation, is influenced by dietary citrate-rich foods. These observations align with previous studies linking dietary patterns to stone risks [24]. Therefore, interpreting urinary output within the context of dietary input can enhance the clinical utility of 24-h urine analyses, enabling targeted dietary modifications to mitigate stone recurrence risk.

The Random Forest and XGBoost models identified urinary calcium and magnesium as key predictors for kidney and multiple location stones, respectively. Elevated urinary calcium levels (OR = 1.1036) have been shown to increase supersaturation, which promotes calcium oxalate and calcium phosphate crystal formation, a known pathway for nephrolithiasis. Similarly, urinary magnesium (OR = 1.1950) has been linked to stone formation, possibly due to its interaction with urinary phosphate. The model’s reliance on these variables underscores their role not only as risk markers but also as potential therapeutic targets. Clinically, reducing dietary calcium intake or enhancing magnesium supplementation may be considered strategies to reduce stone recurrence in high-risk patients, aligning with the model’s predictions.

This study utilized various statistical methods (stepwise regression, Lasso, XGBoost, Random Forest, Super Learner) to identify significant risk factors, ensuring consistency across different modeling approaches. The ensemble method (Super Learner) provided stable performance, with Random Forest showing the highest AUCs, indicating reliable predictive capability. Specifically, using multiple machine learning techniques (XGBoost, Random Forest, and Super Learner), we established models with satisfactory performance to predict the risks of urinary stones at different locations (with AUC-ROC > 0.75 for all stone locations), allowing us to adopt this model as a screening tool in our clinical setting. Achieving an AUC-ROC of >0.75 has also been demonstrated as a screening purpose in other independent urine metabolites profile studies [28]. The machine learning screening tools provide applications in clinical workflows in multiple aspects. First, in our routine clinical practice, 24-h urinary metabolite evaluations can be processed quickly using these machine learning models, allowing physicians to receive immediate predictions regarding a patient’s risk of urinary stone formation. Second, we will explore how these predictive models can be integrated into existing Electronic Health Records (EHRs) or Clinical Decision Support Systems (CDSSs). By embedding these models, clinicians could receive real-time predictions directly within their workflow, aiding in individualized treatment decisions based on a patient’s unique metabolic profile.

There are several limitations of this study. As a cross-sectional study, our research is limited to identifying correlations between risk factors and urinary stones rather than establishing causality as in cohort studies. We focus on the stone location as our response for our classification, which is an important indication for stone characterizations, such as stone size and stability; however, stone location can change over time. Stone composition is also very important in understanding pathophysiology. Under our current circumstances, patient stone specimens and stone composition analysis reports are not well maintained. The collection of stone composition data and including it in the classification study will be an important future work. In addition, some unmeasured confounding variables may affect the classification results. For example, we use urinary sodium excretion to reflect dietary sodium consumption, which is a common approach; however, some other factors (i.e., medications or kidney function) may also affect urinary sodium excretion. Including more patient information from the EHR data will be beneficial. Furthermore, the sample size for our current dataset is relatively small for more complicated machine learning models. We are continuing to collect data, and we will seek external data collaborations in the future to improve the building, validation, and generalizability of our model.

Our future work will focus on further improving the predictive performances of the model to establish an early diagnostic tool (with targeted AUC-ROC > 0.85) in our clinical setting; the targeted AUC-ROC is supported by other independent studies [29,30]. To achieve such goals, in the next stage of our data collection, we aim to increase our sample size to more than 2000 to implement more sophisticated machine learning models, such as artificial neural networks based on the sample size calculations from other independent clinical research [31,32,33]. We will promote data collection and documentation on the urinary metabolic profiles of high-risk patients not only within our clinic but also our collaborative community clinics. We will further evaluate the clinical benefits from implementing these models in terms of improved diagnostic accuracy, reduced diagnosis time, and the ability to develop personalized treatment plans. Early detection and individualized interventions could help prevent stone recurrence, reduce the need for invasive procedures, and ultimately enhance patient care. With routine documentations on urinary metabolic profiles, longitudinal studies will be performed to establish causative links and evaluate the effectiveness of targeted interventions based on these risk factors.

## 5. Conclusions

In conclusion, this study demonstrates the potential of advanced machine learning techniques, particularly the Random Forest algorithm, in identifying significant biomarkers for urolithiasis from 24-h urinary metabolic data. By achieving high predictive accuracy for kidney, ureter, and multiple location stones, we established a machine learning-based screening tool for urinary stones. The findings suggest that specific urinary metabolites, such as magnesium and calcium, play crucial roles in stone formation, while others like creatinine and citrate may offer protective effects. These insights provide a promising foundation for developing personalized prevention strategies, integrating dietary and pharmacological interventions, and advancing the management of urolithiasis in clinical practice.

## Figures and Tables

**Table 1:** The confusion matrix for evaluation of model performance.

		Patients (Kidney/Ureter/Multiple Location Tones)	
		Yes	No
Prediction	Yes	True positive, TP	False positive, FP
	No	False negative, FN	True negative, TN

**Table 2:** Patient description information for kidney stone patients (N = 435).

Candidate Features ^1^	N 287	Y 148	*p*-Value ^2^	Missing Rate ^3^
Gender			0.2363	0
Female	89 (31.01)	55 (37.16)		
Male	198 (68.99)	93 (62.84)		
HBP			0.0158	0
N	205 (71.43)	88 (59.46)		
Y	82 (28.57)	60 (40.54)		
High cholesterol			0.6563	38 (8.74)
N	99 (34.49)	63 (42.57)		
Y	150 (52.26)	85 (57.43)		
Diabetes			0.5144	99 (22.76)
N	188 (65.51)	115 (77.7)		
Y	18 (6.27)	15 (10.14)		
Hyperuricemia			0.7894	38 (8.74)
N	145 (50.52)	89 (60.14)		
Y	104 (36.24)	59 (39.86)		
Hyperparathyroidism			0.6318	89 (20.46)
N	207 (72.13)	134 (90.54)		
Y	2 (0.7)	3 (2.03)		
Age	43.8 (12.78)	43 (14.75)	0.6219	88 (20.23)
BMI	25.5 (3.85)	25.1 (3.64)	0.2631	38 (8.74)
Uric acid	360.3 (96.51)	364.6 (107.54)	0.6860	38 (8.74)
eGFR	88.2 (27.83)	92.9 (25.41)	0.1106	105 (24.14)
Urinary oxalate (24H)	256.3 (199.41)	235.9 (217.52)	0.4235	109 (25.06)
Urinary citrate (24H)	2009.6 (1375.96)	1628.5 (1673.73)	0.0475 *	109 (25.06)
Urinary calcium (24H)	5.7 (3.25)	5.9 (3.58)	0.4314	38 (8.74)
Urinary uric acid (24H)	4.2 (1.54)	4 (1.53)	0.3656	38 (8.74)
Daily sodium intake	5.8 (2.93)	5.2 (2.63)	0.0247 *	38 (8.74)
Urinary creatinine (24H)	14.9 (5.29)	13.8 (4.97)	0.0339 *	38 (8.74)
Urinary phosphorus (24H)	23.4 (8.18)	25.3 (10.66)	0.0759	55 (12.64)
Urinary magnesium (24H)	4.1 (1.66)	4.7 (1.99)	0.0035 *	55 (12.64)
Urine PH (24H)	6.3 (0.71)	6.3 (0.58)	0.8483	26 (5.98)
Urine volume (24H)	2.3 (0.92)	2.4 (0.94)	0.939	55 (12.64)

^1^ Continuous features are represented as mean (SD); categorical features are represented as n (%). ^2^ Group differences were tested using the Chi-square test for categorical features and the *t*-test for the continuous features; * indicates statistical significance (*p* < 0.05). ^3^ Missing rates are represented as n (%).

**Table 3:** Mean performance metrics (based on the 5 imputed datasets) for kidney stones, ureter stones, and multiple location stones prediction models after multiple imputation.

**Kidney Stones**					
**Method**	**Accuracy**	**Precision**	**Recall**	**F1-Score**	**AUC**
GLM	0.605	0.588	0.877	0.704	0.639
Stepwise GLM	0.592	0.578	0.879	0.697	0.639
Lasso	0.551	0.545	0.962	0.696	0.623
XGBoost	0.644	0.617	0.887	0.727	0.780
Random Forest	0.641	0.609	0.915	0.732	0.809
Super Learner	0.656	0.627	0.882	0.733	0.798
**Ureter Stones**					
GLM	0.727	0.663	0.889	0.76	0.79
Stepwise GLM	0.731	0.667	0.889	0.762	0.792
Lasso	0.721	0.653	0.911	0.76	0.791
XGBoost	0.909	0.855	0.979	0.913	0.988
Random Forest	0.876	0.799	0.996	0.886	0.99
Super Learner	0.892	0.825	0.987	0.899	0.985
**Multiple Location Stones**					
GLM	0.595	0.584	0.864	0.696	0.617
Stepwise GLM	0.6	0.585	0.889	0.705	0.63
Lasso	0.573	0.56	0.965	0.709	0.632
XGBoost	0.634	0.609	0.904	0.727	0.75
Random Forest	0.608	0.586	0.929	0.718	0.775
Super Learner	0.62	0.593	0.936	0.726	0.775

**Table 4:** Logistic regression using the top-10 important risk factors identified by Random Forest for kidney stones.

Candidate Features	Parameter Estimate	OR (95% CI)	*p*-Value
Urinary magnesium (24H)	0.1782	1.195 (1.06–1.3525)	0.0041 **
Urinary citrate (24H)	0.0002	0.9998 (0.9997–0.9999)	0.0144 *
Urinary oxalate (24H)	0.0002	1.0002 (0.9991–1.0013)	0.7206
Daily sodium intake	−0.0998	0.9051 (0.8398–0.9731)	0.0078 **
Urinary creatinine (24H)	−0.0479	0.9533 (0.9117–0.996)	0.0336 *
Urinary phosphorus (24H)	0.0167	1.0168 (0.9934–1.0412)	0.1623
eGFR	0.006	1.006 (0.9985–1.0136)	0.1194
Urine volume (24H)	−0.1256	0.882 (0.7151–1.0852)	0.2369
BMI	0.0328	1.0333 (0.9765–1.0939)	0.2569
Urinary calcium (24H)	0.0986	1.1036 (1.0373–1.1757)	0.002 **

** means statistically significant (i.e., *p*-value < 0.05); * means strong statistically significant (i.e., *p*-value < 0.01).

**Table 5:** Logistic regression using the top-10 important risk factors identified by Random Forest for ureter stones.

Candidate Features	Parameter Estimate	OR (95% CI)	*p*-Value
Urinary magnesium (24H)	0.0386	1.0393 (0.9295–1.1618)	0.4968
Urinary citrate (24H)	0.0001	1.0001 (0.9999–1.0002)	0.45
Urine volume (24H)	0.3254	1.3846 (1.1598–1.658)	0.0004 **
eGFR	0.0144	1.0145 (1.0084–1.0209)	0.007 **
Urinary phosphorus (24H)	−0.0065	0.9935 (0.9724–1.0151)	0.5526
Urinary calcium (24H)	0.1425	1.1532 (1.0905–1.221)	0.04 *
Uric acid	0.0049	1.0049 (1.0028–1.007)	<0.001 **
Urinary oxalate (24H)	0.0016	1.0016 (1.0007–1.0026)	0.0008 **
Urinary creatinine (24H)	−0.1541	0.8572 (0.8182–0.8959)	<0.001 **
BMI	0.0792	1.0824 (1.0249–1.1441)	0.0047 **

** means statistically significant (i.e., *p*-value < 0.05); * means strong statistically significant (i.e., *p*-value < 0.01).

**Table 6:** Logistic regression using the top-10 important risk factors identified by Random Forest for multiple location stones.

Candidate Features	Parameter Estimate	OR (95% CI)	*p*-Value
Urinary magnesium (24H)	0.282	1.3258 (1.1814–1.4949)	<0.001 **
Urinary oxalate (24H)	0.0001	0.9999 (0.9989–1.0009)	0.7841
eGFR	0.0147	1.0148 (1.0077–1.0223)	<0.001 **
Urinary citrate (24H)	−0.0002	0.9998 (0.9997–0.9999)	0.0249 *
Urinary phosphorus (24H)	−0.002	0.998 (0.9758–1.0208)	0.863
Urinary creatinine (24H)	−0.0363	0.9644 (0.9168–1.0138)	0.1563
BMI	0.0411	1.0419 (0.9842–1.1036)	0.159
Urine volume (24H)	−0.0418	0.9591 (0.796–1.155)	0.6586
Uric acid	0.0018	1.0018 (0.9998–1.0038)	0.0779
Urinary uric acid (24H)	−0.008	0.992 (0.8452–1.1656)	0.9219

** means statistically significant (i.e., *p*-value < 0.05); * means strong statistically significant (i.e., *p*-value < 0.01).

**Table 7:** Mean performance metrics for kidney stones, ureter stones, and multiple location stones prediction models after multiple imputation (with interaction).

**Kidney Stones**					
Method	Accuracy	Precision	Recall	F1-Score	AUC
XGBoost	0.689	0.745	0.509	0.601	0.792
Random Forest	0.664	0.774	0.394	0.517	0.791
Super Learner	0.684	0.784	0.447	0.565	0.796
**Ureter Stones**					
Method	Accuracy	Precision	Recall	F1-Score	AUC
XGBoost	0.94	0.967	0.916	0.94	0.992
Random Forest	0.936	0.985	0.89	0.935	0.993
Super Learner	0.932	0.99	0.878	0.93	0.991
**Multiple Location Stones**					
Method	Accuracy	Precision	Recall	F1-Score	AUC
XGBoost	0.652	0.722	0.395	0.503	0.776
Random Forest	0.618	0.754	0.26	0.386	0.8
Super Learner	0.659	0.751	0.389	0.504	0.802

**Table 8:** Logistic regression using the top−10 important risk factors identified by Random Forest for kidney stones after removing urinary magnesium (24H).

Candidate Features	Parameter Estimate	OR (95% CI)	*p*-Value
Urinary citrate (24H)	−0.0002	0.9998 (0.9997–0.9999)	0.0307 *
Urinary oxalate (24H)	−0.0007	0.9993 (0.9982–1.0003)	0.1717
Urine volume (24H)	−0.0641	0.9379 (0.7641–1.1503)	−0.5384
Urinary creatinine (24H)	−0.0535	0.9479 (0.8989–0.9986)	0.0458 *
Uric acid	0.0018	1.0018 (0.9997–1.0039)	0.0933
eGFR	0.0091	1.0091 (1.0015–1.0171)	0.0205 *
Urinary phosphorus (24H)	0.0328	1.0334 (1.0117–1.0562)	0.0028 **
Daily sodium intake	−0.0851	0.9184 (0.8546–0.9846)	0.0182 *
Urinary uric acid (24H)	0.0779	1.081 (0.9168–1.2783)	0.3569
BMI	0.0269	1.0272 (0.9688–1.0893)	0.3683

** means statistically significant (i.e., *p*-value < 0.05); * means strong statistically significant (i.e., *p*-value < 0.01).

**Table 9:** Logistic regression using the top-10 important risk factors identified by Random Forest for ureter stones after removing urinary magnesium (24H).

Candidate Features	Parameter Estimate	OR (95% CI)	*p*-Value
Urinary citrate (24H)	−3.277 × 10^−6^	1 (0.9998–1.0001)	0.966
Uric acid	0.0036	1.0036 (1.0016–1.0057)	0.0006 **
Urinary calcium (24H)	0.1534	1.1657 (1.0989–1.2384)	<0.0001 **
Urinary phosphorus (24H)	−0.0161	0.9841 (0.9644–1.0039)	0.1158
eGFR	0.0204	1.0206 (1.0139–1.0279)	<0.0001 **
Urine volume (24H)	0.4956	1.6415 (1.3459–2.0137)	<0.0001 **
Urinary oxalate (24H)	2.00 × 10^−4^	1.0002 (0.9993–1.0011)	0.6659
BMI	0.076	1.079 (1.0219–1.1399)	0.0063 **
Urinary creatinine (24H)	−0.137	0.872 (0.8345–0.9092)	<0.0001 **
Urine PH (24H)	−0.6442	0.5251 (0.3892–0.7002)	<0.0001 **

** means statistically significant (i.e., *p*-value < 0.05).

**Table 10:** Logistic regression using the top-10 important risk factors identified by Random Forest for multiple location stones after removing urinary magnesium (24H).

Candidate Features	Parameter Estimate	OR (95% CI)	*p*-Value
Urinary citrate (24H)	−0.0003	0.9997 (0.9996–0.9999)	0.0004 **
Urinary oxalate (24H)	0.0009	1.0009 (0.9998–1.0019)	0.098
Urine volume (24H)	−0.0415	0.9594 (0.796–1.1555)	0.6617
Urinary phosphorus (24H)	0.0205	1.0208 (1.0007–1.0417)	0.0441 *
BMI	0.0391	1.0399 (0.9831–1.1004)	0.1732
Urinary creatinine (24H)	−0.0686	0.9337 (0.8862–0.9829)	0.0093 **
Uric acid	0.0025	1.0025 (1.0004–1.0046)	0.0182 *
eGFR	0.0122	1.0123 (1.005–1.0200)	0.0012 **
Urinary calcium (24H)	0.1403	1.1507 (1.0813–1.2267)	<0.0001 **
Urinary uric acid (24H)	0.0769	0.9260 (0.788–1.0871)	0.3473

** means statistically significant (i.e., *p*-value < 0.05); * means strong statistically significant (i.e., *p*-value < 0.01).

## Data Availability

Analytical data and software are available upon request to the corresponding authors.

## Supplementary Materials

The following supporting information can be downloaded at: https://www.mdpi.com/article/10.3390/metabo14120674/s1, Figure S1: Correlation matrix among continuous candidate features; Figure S2: Importance plots from XGBoost of (a) kidney stones, (b) ureter stones, and (c) multiple location stones. Figure S3: SHAP plot by Random Forest (left) and XGBoost (right) for ureter stones; Figure S4: SHAP plot by Random Forest (left) and XGBoost (right) for multiple location stones; Figure S5: Model performance among machine learning techniques for ureter stones; Figure S6: Model performance among machine learning techniques for multiple location stones. Table S1: Patient description information for ureter stone patients (N = 435); Table S2: Patient description information for multiple location stone patients.
