# Supplementary material for: Machine Learning Models Decoding the Association Between Urinary Stone Diseases and Metabolic Urinary Profiles
Source: Metabolites. 2024 Dec 3;14(12):674. doi: 10.3390/metabo14120674 (PMC11678200; doi:10.3390/metabo14120674)
Supplement: Supplementary file 1 [file metabolites-14-00674-s001.zip › metabolites-3196185-supplementary.pdf]

Supplementary material for "Machine Learning Models Decoding the Association Between Urinary Stone Diseases and Metabolic Urinary Profiles"

In this section, we show the correlation plot among continuous candidate features (Figure S1) and patient description information for Ureter Stone (Table S1) and Multiple Location Stones (Table S2). Figure S1 indicates that eGFR and Serum Creatinine have a strong negative correlation (close to -1). Therefore, we delete Serum Creatinine in our analysis. Table S1 shows that diabetes is the only significant factor for Ureter Stone. Table S2 shows that eGFR and Urinary Magnesium (24H) have significant association with Multiple Location Stones.

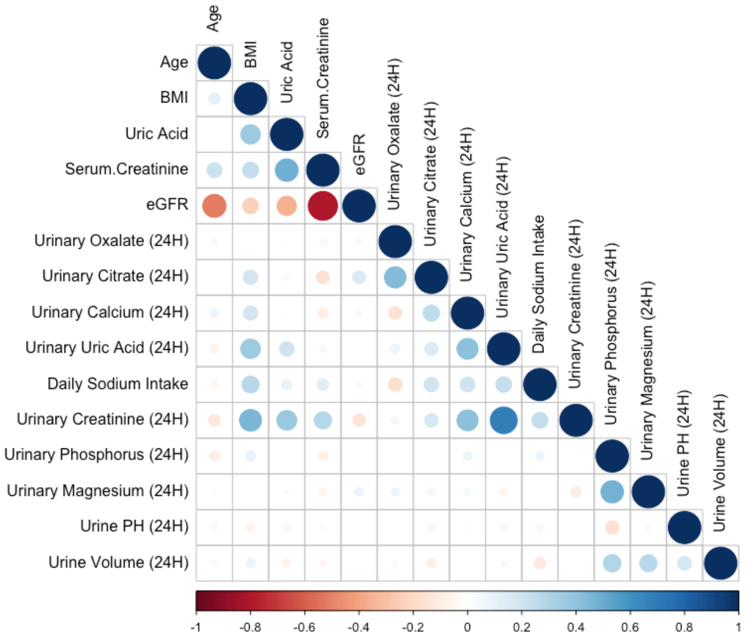

Figure S1. Correlation Matrix among continuous candidate features

Table S1. Patient description information for Ureter Stone (N=435)

Candidate Features[1]	N 401	Y 34	P-value[2]	Missing Rate[3]
Gender			0.6366	0
Female	131(32.67)	13(38.24)		
Male	270(67.33)	21(61.76)		
HBP			0.3604	0
N	273(68.08)	20(58.82)		
Y	128(31.92)	14(41.18)		
High Cholesterol			1	38(8.74)
N	148(36.91)	14(41.18)		
Y	215(53.62)	20(58.82)		
Diabetes			0.0171*	99(22.76)

N	281(70.07)	22(64.71)		
Y	26(6.48)	7(20.59)		
Hyperuricemia			0.8438	38(8.74)
N	215(53.62)	19(55.88)		
Y	148(36.91)	15(44.12)		
Hyperparathyroidism			0.9534	89(20.46)
N	310(77.31)	31(91.18)		
Y	4(1)	1(2.94)		
Age	43.5(13.14)	43.4(17.58)	0.9718	88(20.23)
BMI	25.3(3.75)	25.4(4.05)	0.9688	38(8.74)
Uric Acid	362.2(101.18)	358.2(96.16)	0.816	38(8.74)
eGFR	89.3(24.72)	98.3(41.85)	0.2401	105(24.14)
Urinary Oxalate (24H)	247.4(202)	286.8(245.45)	0.4692	109(25.06)
Urinary Citrate (24H)	1881.1(1490.39)	2052.8(1375.62)	0.5792	109(25.06)
Urinary Calcium (24H)	5.7(3.36)	6.4(3.53)	0.2799	38(8.74)
Urinary Uric Acid (24H)	4.1(1.55)	4(1.45)	0.8157	38(8.74)
Daily Sodium Intake	5.5(2.79)	6(3.38)	0.4652	38(8.74)
Urinary Creatinine (24H)	14.6(5.13)	13.2(5.77)	0.1775	38(8.74)
Urinary Phosphorus (24H)	24.1(9.18)	23.7(8.98)	0.8105	55(12.64)
Urinary Magnesium (24H)	4.3(1.84)	4.5(1.24)	0.3072	55(12.64)
Urine PH (24H)	6.3(0.67)	6.2(0.64)	0.2441	26(5.98)
Urine Volume (24H)	2.3(0.91)	2.6(1.06)	0.2599	55(12.64)

[1]Continuous features are represented as Mean (SD), categorical features are represented as n (%).
[2]Group differences were tested using Chi-square test for categorical features, t-test for the continuous features;* indicates statatistically signifcant ($p<0.05$).
[3]Missing rate are represented as n(%).

Table S2. Patient description information for Multiple Location Stones

Candidate Features[1]	N<br>305	Y<br>163	P-value[2]	Missing Rate[3]
Gender			0.4173	0
Female	94(30.82)	57(34.97)		
Male	211(69.18)	106(65.03)		
HBP			0.0567	0
N	215(70.49)	100(61.35)		
Y	90(29.51)	63(38.65)		
High Cholesterol			0.8378	71(15.17)
N	94(30.82)	68(41.72)		
Y	140(45.9)	95(58.28)		
Diabetes			0.3545	131(27.99)
N	178(58.36)	126(77.3)		
Y	16(5.25)	17(10.43)		
Hyperuricemia			0.905	71(15.17)
N	139(45.57)	95(58.28)		
Y	95(31.15)	68(41.72)		
Hyperparathyroidism			0.7683	121(25.85)

N	194(63.61)	148(90.8)		
Y	2(0.66)	3(1.84)		
Age	43.9(12.37)	42.9(15.04)	0.5178	120(25.64)
BMI	25.5(3.91)	25.1(3.64)	0.377	45(9.62)
Uric Acid	361.3(95.6)	364.6(105.64)	0.752	45(9.62)
eGFR	87(24.23)	94(29.29)	0.0191*	137(29.27)
Urinary Oxalate (24H)	257.7(200.67)	234.9(213.42)	0.3534	142(30.34)
Urinary Citrate (24H)	1998.9(1385.21)	1684.1(1641.27)	0.0865	142(30.34)
Urinary Calcium (24H)	5.5(3.24)	6.1(3.57)	0.1216	65(13.89)
Urinary Uric Acid (24H)	4.1(1.55)	4(1.53)	0.4093	65(13.89)
Daily Sodium Intake	5.8(3.05)	5.4(2.79)	0.215	65(13.89)
Urinary Creatinine (24H)	14.8(5.24)	13.9(5.14)	0.0695	65(13.89)
Urinary Phosphorus (24H)	23.4(8.26)	25.3(10.4)	0.0655	65(13.89)
Urinary Magnesium (24H)	4(1.68)	4.7(1.93)	0.0012*	65(13.89)
Urine PH (24H)	6.3(0.72)	6.3(0.59)	0.7602	29(6.2)
Urine Volume (24H)	2.4(1)	2.4(0.94)	0.9256	65(13.89)

[1]Continuous features are represented as Mean (SD), categorical features are represented as n (%).

[2]Group differences were tested using Chi-square test for categorical features, t-test for the continuous features;* indicates statatistically signifcant ($p<0.05$).

[3]Missing rate are represented as n(%).

This section shows the importance plot for XGBoost (Figure S2) and SHAP from Random Forests and XGBoost for Ureter Stone and Multiple Location Stones (Figure S3, S4) patients.

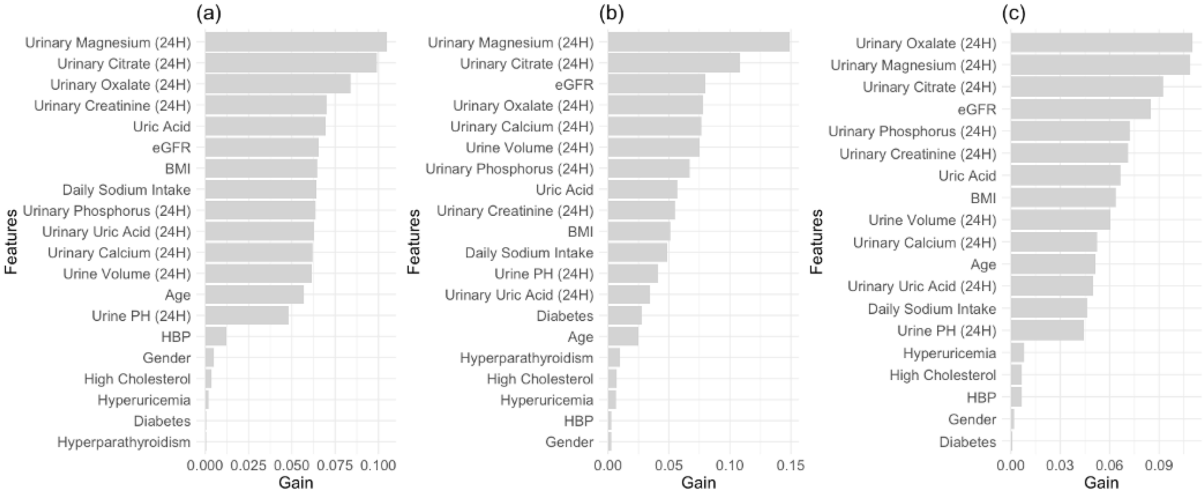

Figure S2. Importance plots from XGBoost of (a) Kidney Stone, (b) Ureter Stone, (c) Multiple Location Stones: The importance of each variable is measured by the Mean Decrease in from one of the five imputed datasets during the multiple imputation.

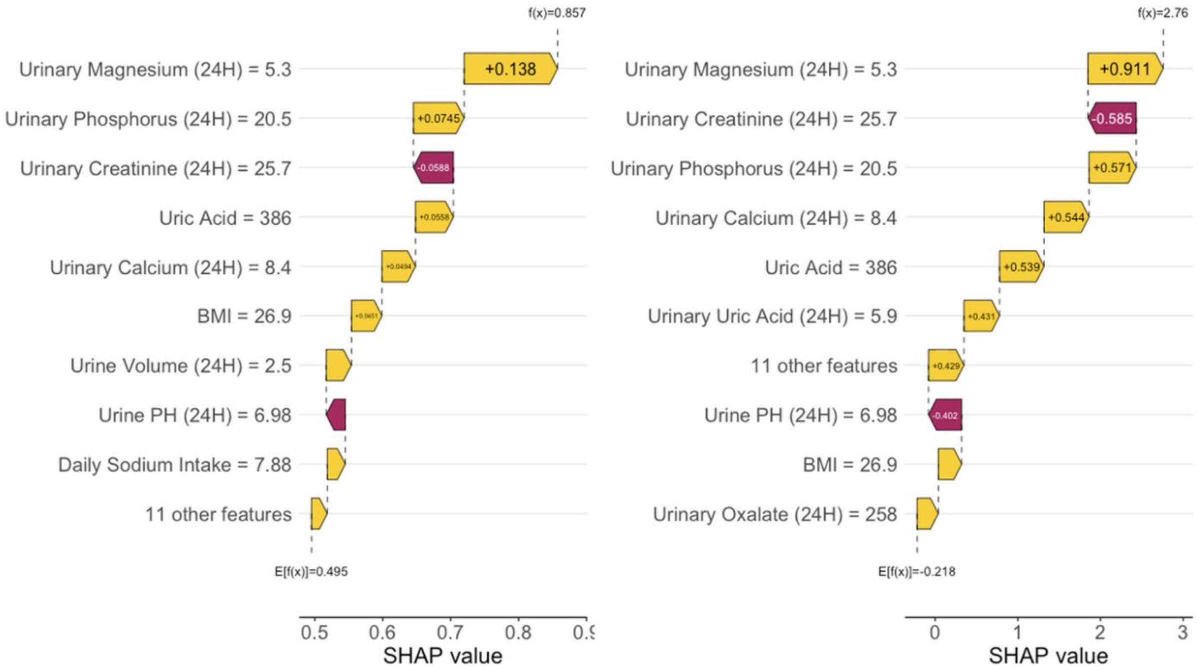

Figure S3. SHAP plots by Random Forest (left) and XGBoost (right) for predicting Ureter stone risk: the SHAP values are calculated from one of the five imputed datasets during the multiple imputation.

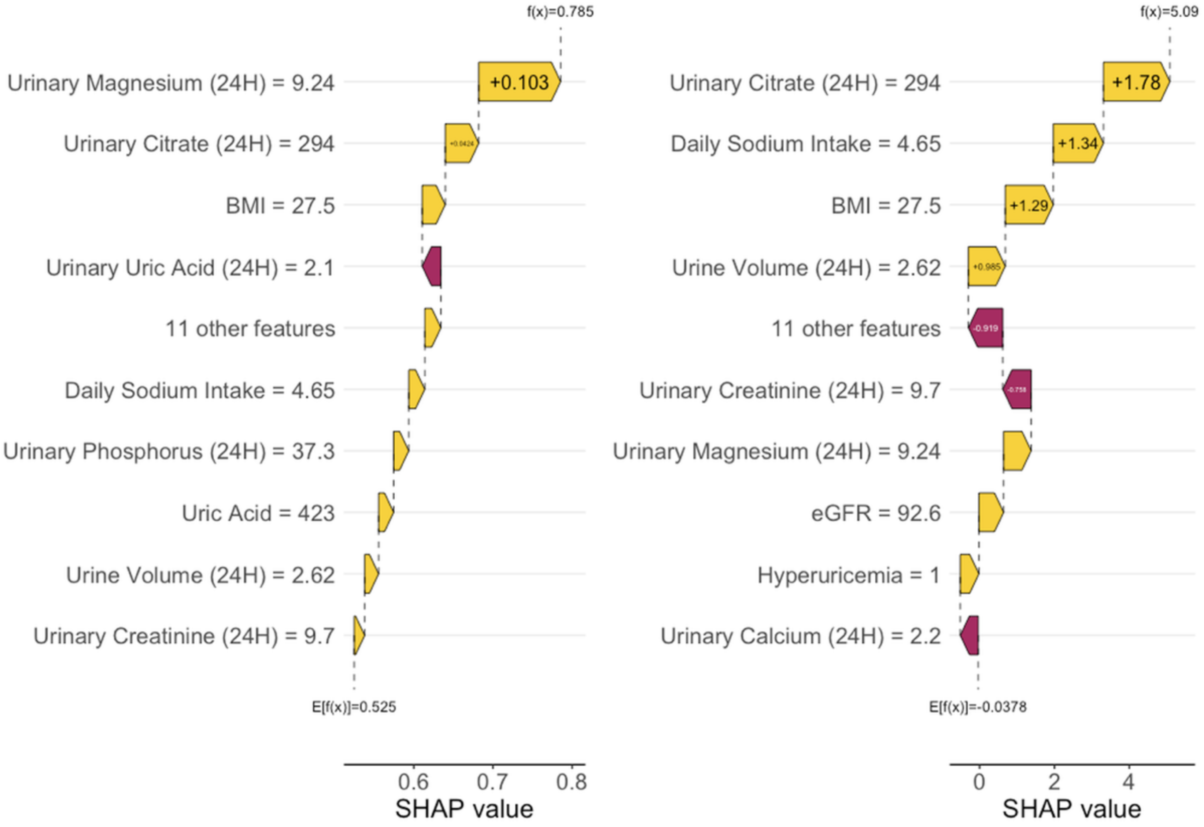

Figure S4. SHAP plots by Random Forest (left) and XGBoost (right) for predicting Multiple Location Stones risk: the SHAP values are calculated from one of the five imputed datasets during the multiple imputation.

The model performances for Ureter Stone (Figure S5) and Multiple location stones (Figure S6) are also shown in this section.

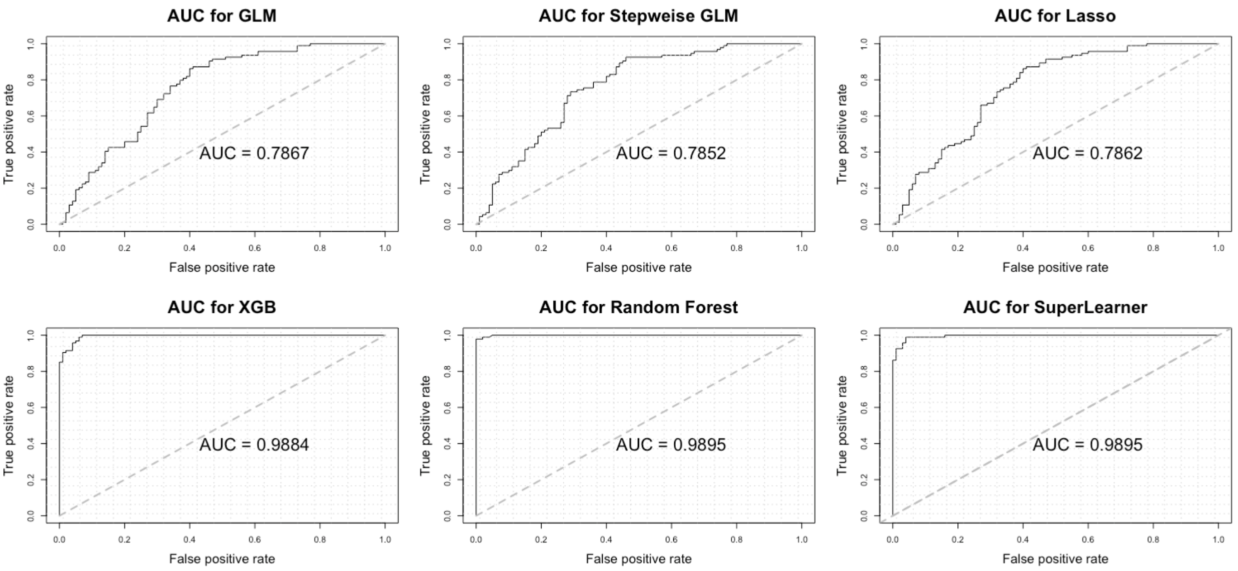

Figure S5. ROC curve among machine learning techniques (based on the 5 imputed datasets) for the Ureter Stone.

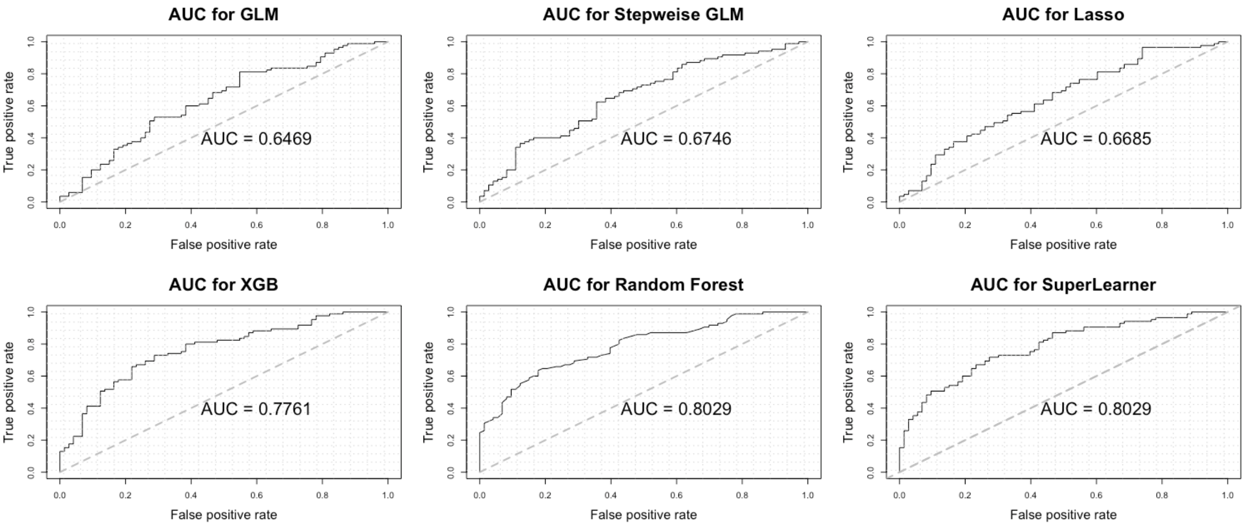

Figure S6. ROC curve among machine learning techniques (based on the 5 imputed datasets) for the Multiple Location Stones.
